# Estimation of the risk for radiation-induced liver disease following photon- or proton-beam radiosurgery of liver metastases

**DOI:** 10.1186/s13014-018-1151-6

**Published:** 2018-10-22

**Authors:** Gracinda Mondlane, Ana Ureba, Michael Gubanski, P A Lind, Albert Siegbahn

**Affiliations:** 1 Department of Physics – Medical Radiation Physics, Stockholm University, Stockholm, Sweden; 2 Department of Physics, Universidade Eduardo Mondlane, Maputo, Mozambique; 3 Department of Oncology and Pathology, Karolinska Institutet, Stockholm, Sweden; 4 Department of Oncology and Pathology, Karolinska University Hospital, Stockholm, Sweden; 5 Department of Oncology, Södersjukhuset, Stockholm, Sweden

**Keywords:** RILD, Liver metastases, SBRT, IMPT, Patient selection

## Abstract

**Background:**

Radiotherapy of liver metastases is commonly being performed with photon-beam based stereotactic body radiation therapy (SBRT). The high risk for radiation-induced liver disease (RILD) is a limiting factor in these treatments. The use of proton-beam based SBRT could potentially improve the sparing of the healthy part of the liver. The aim of this study was to use estimations of normal tissue complication probability (NTCP) to identify liver-metastases patients that could benefit from being treated with intensity-modulated proton therapy (IMPT), based on the reduction of the risk for RILD.

**Methods:**

Ten liver metastases patients, previously treated with photon-beam based SBRT, were retrospectively planned with IMPT. A CTV-based robust optimisation (accounting for setup and range uncertainties), combined with a PTV-based conventional optimisation, was performed. A robustness criterion was defined for the CTV (V_95%_ > 98% for at least 10 of the 12 simulated scenarios). The NTCP was estimated for different endpoints using the Lyman-Kutcher-Burman model. The ΔNTCP (*NTCP*_*IMPT*_ − *NTCP*_*SBRT*_) for RILD was registered for each patient. The patients for which the NTCP (RILD) < 5% were also identified. A generic relative biological effectiveness of 1.1 was assumed for the proton beams.

**Results:**

For all patients, the objectives set for the PTV and the robustness criterion set for the CTV were fulfilled with the IMPT plans. An improved sparing of the healthy part of the liver, right kidney, lungs, spinal cord and the skin was achieved with the IMPT plans, compared to the SBRT plans. Mean liver doses larger than the threshold value of 32 Gy led to NTCP values for RILD exceeding 5% (7 patients with SBRT and 3 patients with the IMPT plans). ΔNTCP values (RILD) ranging between − 98% and − 17% (7 patients) and between 0 and 2% (3 patients), were calculated.

**Conclusions:**

In this study, liver metastases patients that could benefit from being treated with IMPT, based on the NTCP reductions, were identified. The clinical implementation of such a model-based approach to select liver metastases patients to proton therapy needs to be made with caution while considering the uncertainties involved in the NTCP estimations.

## Background

Radiotherapy of liver metastases is commonly performed using photon-beam based extracranial radiosurgery, also known as stereotactic body radiation therapy (SBRT) [1]. The radiosensitivity of the liver sets a limit for how high doses that can be delivered. If the recommended threshold doses are exceeded, there will be an increased risk for the development of radiation-induced liver disease (RILD). RILD occurs between 2 weeks and 4 months after the completion of the liver irradiation and is a clinical syndrome of anicteric hepatomegaly, ascites and elevated liver enzymes [2]. However, it has been observed clinically that it is possible to deliver high hypofractionated doses to limited volumes of the liver, while only producing low levels of toxicity [1, 3], owing to the parallel functional organization of the liver [4].

Research and technological progress in recent years have led to the introduction of new radiation treatment techniques, e.g., scanned proton-beam therapy (PBT). The physical characteristics of proton-beam dose deposition allow highly conformal dose distributions to be delivered to the tumour volumes with reduced integral doses given to the healthy tissues, compared to when photon-beams are used [5]. For instance, if PBT is used for the treatment of liver malignancies, the improved dosimetric sparing of the healthy part of the liver could potentially lead to a reduction of the occurrence of RILD. However, the achievable sparing of the healthy part of the liver can be expected to vary from patient to patient depending on the individual anatomy, diagnosis and underlying liver disease/function.

The selection of liver metastases patients for treatment with PBT should be clinically justified in terms of improvements in the dose sparing of the healthy non-targeted part of the liver and a reduction of the corresponding risks for treatment-induced liver toxicity, compared to what can be achieved with photon-beam based SBRT. In addition, treatment decisions are based on different aspects, such as cost-effectiveness. When identifying patients suitable for treatment with either photon or proton therapy, the relatively high cost of proton therapy has to be balanced against the clinical gain in terms of sparing of the OARs [6]. In this context, a method for patient selection, based on comparisons of dosimetric values and of normal-tissue complication probabilities (NTCPs), has been proposed [7]. In a previous study by Mondlane et al. of radiosurgery of liver metastases [8], the potential to reduce the integral doses given to the organs at risk (OARs) with intensity-modulated proton therapy (IMPT) was demonstrated. In the present study, a comparison of the NTCPs calculated for SBRT- or IMPT-based radiosurgery of liver metastases was performed. The aim of this study was to identify patients, for which IMPT could provide a reduction of the risk for RILD, compared to photon-based SBRT.

## Methods

### Patients and delineation of the structures

Ten patients (aged between 66 and 89 years) diagnosed with liver metastases from colorectal-cancer were included in this study. These patients had previously been treated with photon-based SBRT at the Karolinska University Hospital. Information about the patient setup, fractionation schedule, photon dose-delivery mode, tumour location and abdominal compression are shown in Table 1.

**Table 1:** Patient setup and treatment description

*Patient*	*SBRT*	*Fractionation*	*Abdominal compression*	*PTV (cm* ^*3*^ *)*	*CTV (cm* ^*3*^ *)*	*Tumour location (liver segment)*
1	3D-CRT	15 Gy × 3	Yes	59.6	21.3	7
2	3D-CRT	17 Gy × 3	Yes	73.1	21.6	4a
3	VMAT	8 Gy × 7	No	332.3	157.0	7
4	3D-CRT	8 Gy × 5	Yes	302.6	142.3	5
5	3D-CRT	7 Gy × 8	No	66.4	19.8	1
6	VMAT	7 Gy × 8	No	294.1	145.1	8
7	3D-CRT	15 Gy × 3	No	18.6	3.5	4b
8	VMAT	7 Gy × 8	Yes	78.6	23.8	4a
9	3D-CRT	17 Gy × 3	No	30.2	4.7	5
10	3D-CRT	15 Gy × 3	No	72.3	24.8	5

The delineation of the relevant structures was performed on the planning computed tomography (CT) image sets with the Eclipse treatment planning system (TPS) (Varian Medical Systems, Palo Alto, California, Version 13.0, Build 13.0.33). These CT image sets contained 3.0 mm thick slices. The planning target volume (PTV) was created by expansion of the clinical target volume (CTV) with 10 mm in the cranio-caudal direction and 5 mm in the axial plane. Four-dimensional CT (4D-CT) image sets were used to assess the amplitude of the CTV motion. Abdominal compression was used to reduce the target volume motion if the motion amplitude was larger than the CTV-to-PTV margin. The median values of the CTV and PTV were 22.7 cm^3^ (range 3.5–157.0 cm^3^) and 72.7 cm^3^ (range 18.6–332.3 cm^3^), respectively. The OARs considered in this study were the healthy part of the liver, skin, lungs, spinal cord and right kidney. The heathy part of the liver was considered to be the whole liver volume, subtracted with the CTV. The skin and spinal cord structures were delineated in the irradiated region of the body structure. To obtain the skin structure, a 3-mm layer was extracted from the body structure. The lungs and the right kidney were also contoured.

### Treatment planning

The Eclipse TPS was used to prepare both the SBRT and IMPT plans. The SBRT treatments were delivered with 6-MV photon beams generated by a Varian linear accelerator (Varian Medical Systems, Palo Alto, California). The proton beam data used in the IMPT planning was obtained from a facility with an IBA cyclotron (Ion Beam Applications S.A., Louvain-La-Neuve, Belgium), with which proton kinetic energies between 60 and 230 MeV can be used for treatment. A range shifter of water-equivalent thickness of 3.5 g/cm^2^ was used when necessary to ensure target dose-coverage at shallow depths. A generic relative biological effectiveness (RBE) value of 1.1 was assumed for the proton beams.

The SBRT plans, used for the actual treatments, were set as reference plans in the comparison. These SBRT plans had been prepared for three-dimensional conformal radiotherapy (3D-CRT), implemented with co-planar static fields (7 patients) or volumetric-modulated arc therapy (VMAT) performed with two full arcs (3 patients). The VMAT method was used for the patient cases for which the target volume was large, of a more complex shape, and a satisfactory sparing of the healthy part of the liver could otherwise not be achieved. The planning objective for SBRT was set to cover the entire PTV with at least 100% of the prescribed dose. An inhomogeneous dose distribution was allowed inside of the PTV, with maximum doses in the range from 145 to 150% of the prescribed dose. The doses given to the periphery of the CTV in the SBRT plans were registered and used to define the objectives in the IMPT planning.

The IMPT plans were prepared using the selective robust optimisation approach, as described by Li et al. [9], in which a CTV-based robust optimisation is performed, taking both the range and setup uncertainties into consideration. A conventional optimisation for the PTV is also considered in this method, using the same objectives as in the SBRT planning. This approach was chosen to allow for the inhomogeneous target doses typically used in SBRT treatments. A setup uncertainty of 10 mm (cranio-caudal direction) and 5 mm (axial plane), which corresponds to the CTV-to-PTV expansion margins used in the planning for SBRT, was considered as perturbation parameter values in the robust optimisation of the IMPT plans. In addition, a proton range uncertainty of 3.5% was also assumed. In the IMPT planning, an irradiation configuration with two fields was chosen in order to obtain a high degree of target-dose conformity, while sparing as much as possible the surrounding healthy tissues, mainly the healthy part of the liver. When selecting the angles of incidence of the proton beams, the direct irradiation through one of the kidneys and the spinal cord was avoided. The same fixation and immobilisation of the patients were assumed in the planning for IMPT, as for photon-beam SBRT. The stereotactic body frame, which was used for patient fixation during the acquisition of the planning CT image sets and during treatment with SBRT, was assigned the Hounsfield unit value of air in the IMPT planning. This step was taken to avoid introducing additional uncertainties in the proton dose calculation related to the partly unknown material composition of the frame. The normal tissue optimisation constraints used in the SBRT- and IMPT-planning were set as follows (the doses are shown for equivalent 2-Gy per fraction treatments): D_40_ < 24 Gy (IsoE) for the right kidney, D_max_ < 45 Gy (IsoE) for the spinal cord and V_20_ < 20% for the lungs. Efforts were also made to minimise the mean dose given to the healthy part of the liver.

### Dosimetric evaluation

For each patient, a robustness analysis of the IMPT plans was performed based on the robustness criterion defined for the CTV, i.e., D_98_ > 95%, for at least 10 of the 12 simulated scenarios. The target dose coverage in the SBRT and IMPT treatment plans was then evaluated by comparing pairwise the D_2_ and D_98_ values for the CTV and the conformity index (CI) [10] for the PTV. The dosimetric evaluation of the IMPT plans was performed on the nominal plans. The dose volume histograms (DVHs) obtained for the OARs with the two treatment modalities were compared and the liver mean dose was also registered.

### Assessment of NTCP

The assessment of NTCP was done using the Lyman-Kutcher-Burman (LKB) model [11, 12]. The physical doses in the extracted DVHs were converted to equivalent DVHs for 1.5 Gy per-fraction (for the healthy part of the liver) and 2 Gy per-fraction (for the remaining OARs). The LKB-model parameters used and the biological endpoints considered are shown in Table 2. These parameters are the most updated published NTCP parameters and the most clinically relevant for the respective OARs considered. The values of the linear-quadratic (LQ) model parameter *α*/*β* for the different OARs used are also presented in Table 2.

**Table 2:** Values of the LKB-model parameters, the corresponding biological-endpoints and the LQ-model parameter for all OARs

*OAR*	*n*	*m*	*TD*_*50*_ *(Gy)*	*Endpoint*	*α*/*β (Gy)*	*Sources*
Skin	0.38	0.14	39	Radiation-induced skin toxicity	3^a^	Pastore et al. (2016) [31]
Lung	1	0.41	29.9	Symptomatic radiation pneumonitis	3	Semenenko & Li (2008) [32]
Liver	0.97	0.12	45.8	Radiation-induced liver disease (RILD)	2	Dawson et al. (2002) [2]
Spinal cord	0.05	0.175	66.5	Radiation myelitis	0.87^b^	Burman et al. (1991) [33]
Right kidney	0.70	0.10	28	Clinical nephritis	3^a^	Burman et al. (1991) [33]

^a^Values of the assumed *α*/*β* parameter for these OARs; ^b^the value of the *α*/*β* parameter for the spinal cord was taken from Schultheiss (2008) [34]

To identify patients who would benefit from being treated with IMPT, two different approaches were considered. The first approach consisted in determining the difference in the estimated NTCP for RILD for the SBRT and IMPT plans, i.e., the ΔNTCP (*NTCP*_*IMPT*_ − *NTCP*_*SBRT*_) [13]. The second approach was based on accepting patients for IMPT if the NTCP for RILD was lower than SBRT and also below 5%. This threshold NTCP value corresponds to a liver mean dose of 32 Gy for 2 Gy per fraction treatments [4]. It is also the commonly used dosimetric constraint for the liver [14]. The NTCPs obtained for the remaining OARs with the two treatment modalities were also compared.

### Statistical analysis

The obtained dosimetric and NTCP values with the SBRT and IMPT plans, were compared pairwise using a two-sided Wilcoxon signed-rank test. A *p*-value of < 0.05 was considered statistically significant.

## Results

**Fig. 1:**
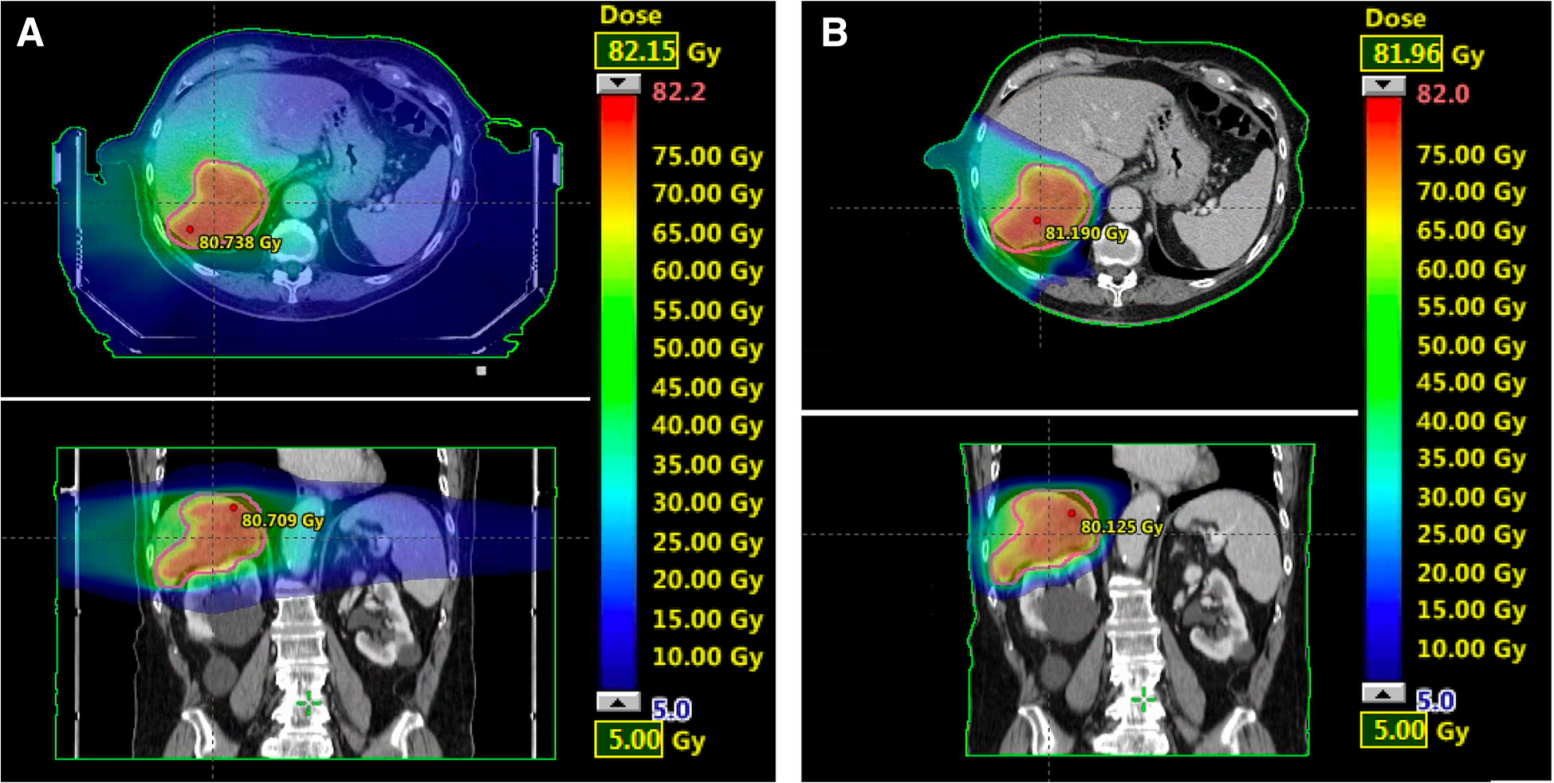
Dose distributions on axial and coronal CT-slices for patient #3 for (**a**) VMAT and (**b**) IMPT

Typical dose distributions obtained with the SBRT and IMPT plans are presented in Fig. 1 for patient #3. The SBRT treatment for this patient was performed with VMAT.

**Fig. 2:**
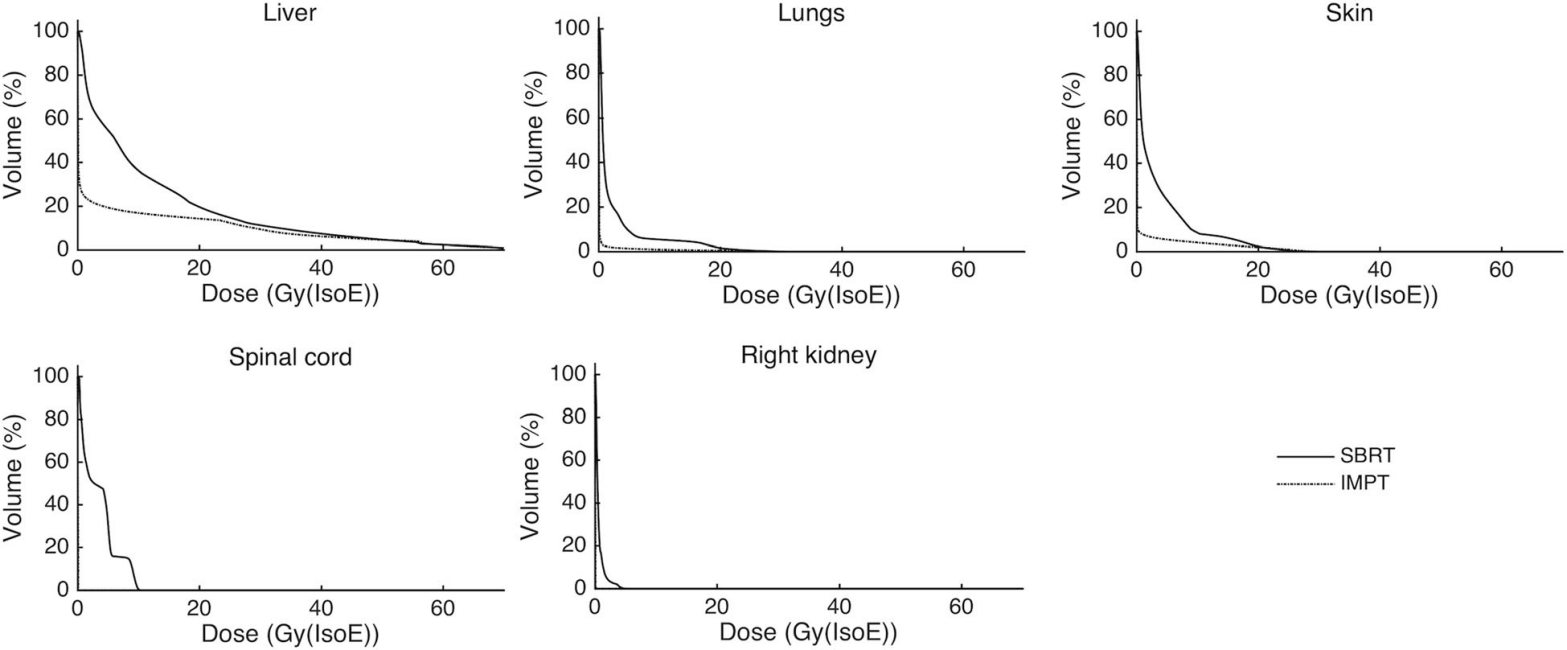
Median DVHs for the OARs with the SBRT- (full lines) and the nominal IMPT-plans (dashed lines)

The robustness criterion set for the CTV was achieved for all patients with the IMPT plans. For the CTV, the median values of the IMPT-to-SBRT ratios of D_2_ and of D_98_ were 1.0 (range 1.0–1.0), *p* > 0.05. For the PTV, a median value of the CI of 1.2 (range 1.0–1.4) was obtained with the SBRT plans and 1.5 (range 1.1–2.3) with the IMPT plans, *p* < 0.05. In general, an improved dosimetric sparing of the OARs was obtained with the IMPT plans, compared to the SBRT plans (Fig. 2). For the particular case of the healthy part of the liver, significantly lower mean doses were registered with IMPT (Table 3).

**Table 3:** Mean doses (2 Gy per-fraction), NTCPs and ΔNTCP for the healthy part of the liver

Patient #	Liver mean dose (Gy (IsoE))		NTCP for RILD (%)		ΔNTCP (%)
	SBRT	IMPT^a^	SBRT	IMPT^a^	
1	38	18 (15–21)	38	0 (0–0)	- 38
2	49	26 (21–30)	98	1 (0–5)	−97
3	60	39 (31–45)	100	53 (5–91)	−47
4	51	33 (30–35)	99	10 (4–16)	- 89
5	30	31 (29–33)	3	5 (1–5)	2
6	39	22 (17–25)	44	0 (0–0)	- 44
7	26	19 (17–20)	0	0 (0–0)	0
8	18	14 (9–18)	0	0 (0–0)	0
9	34	15 (14–16)	17	0 (0–0)	- 17
10	56	40 (36–41)	100	59 (27–73)	- 41

^a^The values in parentheses represent the range of values of the mean doses and NTCPs obtained for the simulated uncertainty scenarios in the IMPT plans

The IMPT plans resulted in a significant reduction of the calculated NTCP for RILD, compared to the SBRT plans (Table 3). The median NTCP for RILD was 41% (range 0 - 100%) with SBRT and 1% (0 - 59%) with IMPT (*p* < 0.05). In the assessment of ΔNTCP for the healthy part of the liver, negative ΔNTCP values (ranging from − 97% to − 17%) were obtained for 7 patients, who would benefit from being treated with IMPT (Table 3). For the remaining 3 patients, ΔNTCP values of 0% and 2% were determined, which indicated that improved plans could not be achieved with IMPT for these patients. Furthermore, NTCP (RILD) values below 5%, which were also lower with IMPT compared to SBRT, were obtained for 4 patients (Table 3). For the remaining patients, SBRT would be the treatment of choice since comparable NTCPs for RILD were obtained with both the SBRT and the IMPT plans (3 patients) and because the NTCPs determined with either the SBRT or IMPT plans were greater than 5% (3 patients).

**Fig. 3:**
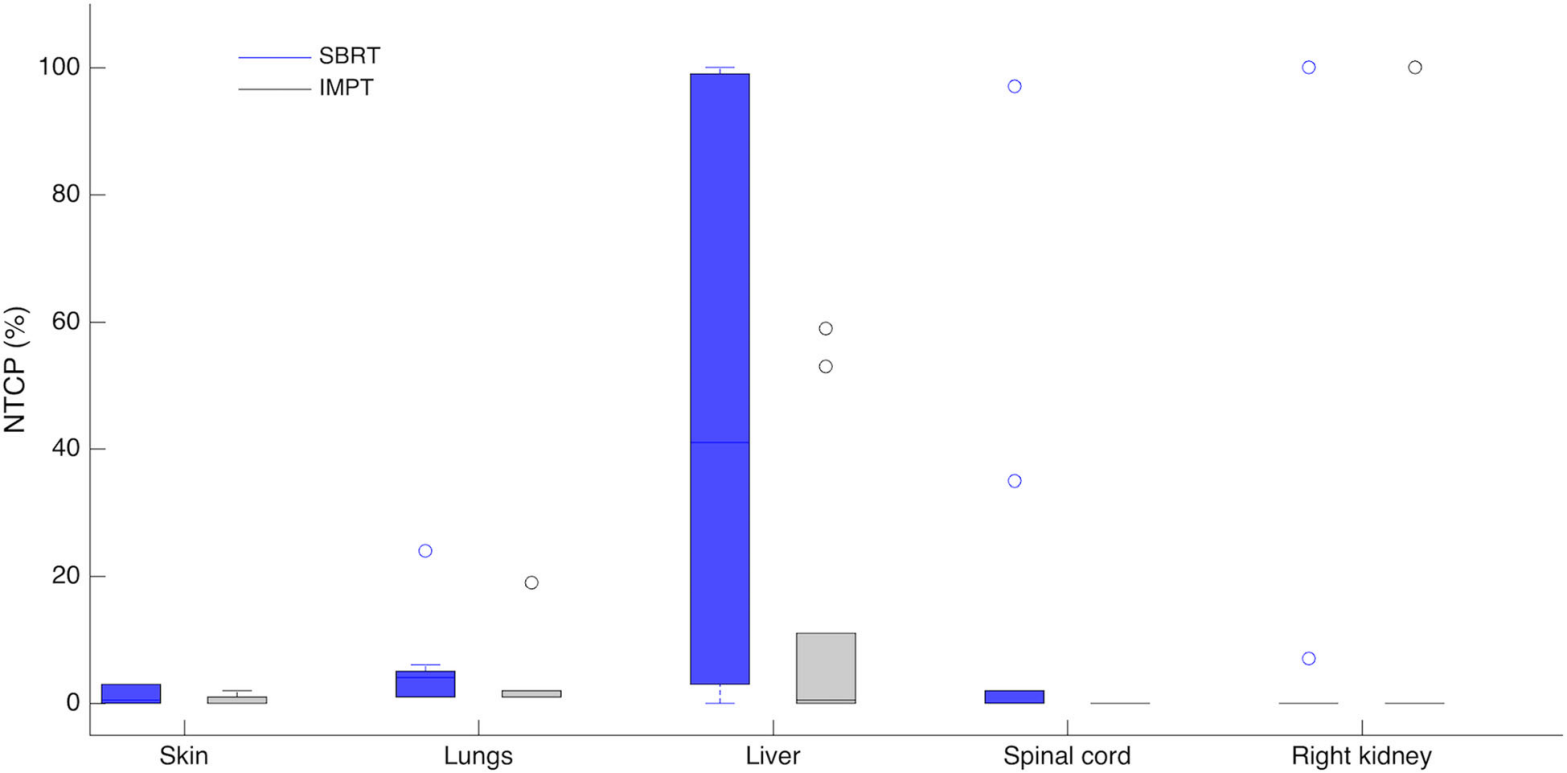
Boxplot showing the calculated NTCPs for all OARs for the SBRT- (blue) and IMPT-plans (grey)

**Fig. 4:**
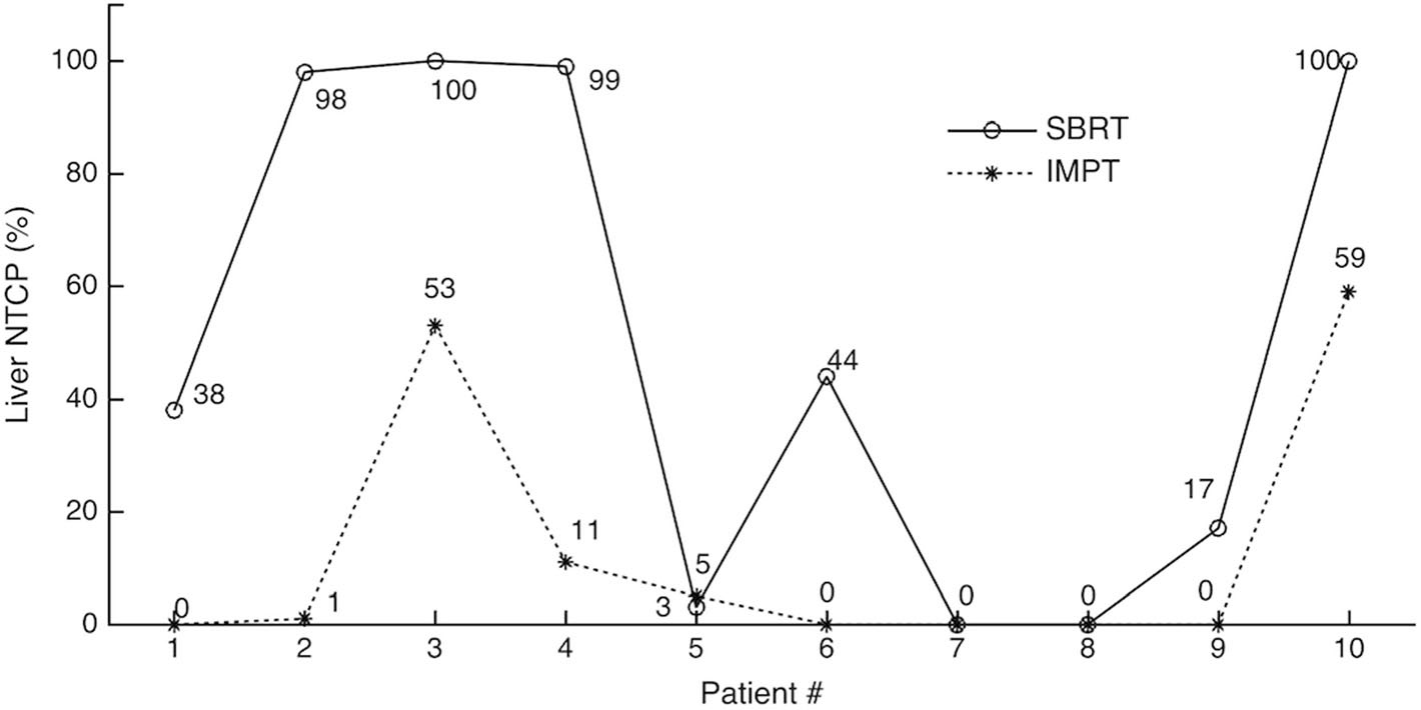
Risk for radiation-induced liver disease obtained with the SBRT and IMPT plans for all patients

A significant decrease in the NTCP values was also registered for the lungs with the IMPT plans (*p* < 0.05). However, for the remaining OARs (right kidney, spinal cord and skin), there was no significant difference between the NTCP values obtained with the SBRT and IMPT plans (*p* > 0.05). A summary of the results obtained in the NTCP assessment is presented in Fig. 3, for all OARs. For the particular case of the healthy part of the liver, the NTCP results obtained with the SBRT and IMPT plans are also shown in Fig. 4.

## Discussions

In the present study, an NTCP-model based approach for selecting liver metastases patients to radiosurgery performed with SBRT or IMPT was evaluated. Patients who would benefit from being treated with IMPT were identified based on the reduction of the NTCP for RILD. We found that the probability of observing RILD was reduced with IMPT for the majority of the patients included in this study. The improved sparing of the surrounding healthy tissue, which was achieved with IMPT also led to a reduction of the NTCP for the lungs, while similar NTCP values were obtained for the remaining OARs, compared to the SBRT plans. These results indicate that the main advantage of IMPT in the treatment of liver tumours is connected to the improved sparing of the healthy part of the liver, which is also known to be the limiting OAR in these treatments.

The advent of new radiotherapy modalities introduces new considerations in the optimal treatment selection process for the individual patients. NTCP-model based approaches for patient selection to photon- or proton-beam therapy, have also been suggested [7, 13]. These models are based on the potential reduction of normal tissue toxicity with PBT, compared to what is achievable with photon beams. In this study, we considered two selection criteria. The first of these was based on the difference in the estimated NTCPs calculated for the IMPT and SBRT plans. According to this criterion, a patient should be selected to be treated with the technique which provided the lowest NTCP. According to the second criterion, patients should only be selected to IMPT if the estimated NTCP for RILD was lower than for SBRT and also below 5%. Based on the first criterion, 70% of the patients included in this study would be selected for IMPT, while with the second criterion, only 40% of the patients would be selected for IMPT. The use of the ΔNTCP approach requires that a threshold value, which is clinically meaningful, is set. However, the reduction of toxicity achievable with proton therapy, which can be translated into a level of clinical gain, is still to be determined for specific tumour sites and their corresponding OARs. Moreover, as proposed by Widder et al. [13], the NTCP-model based estimations should also be validated against the observed toxicity following proton therapy.

The advantages of PBT, compared to photon-beam RT, are in general more enhanced for larger tumour volumes. Photon RT of large tumour volumes involves the irradiation of large volumes of healthy tissue surrounding the target volume. These large irradiated volumes can potentially be reduced with the use of PBT. For smaller tumour volumes, on the other hand, the use of PBT does not lead to considerable improvements. This fact was observed in the present study for three patients (patients #5, 7 and 8) with small PTVs (between 18.6 and 78.6 cm^3^), located in the liver segments 1, 4b and 4a, respectively (Table 1). For these patients, comparable values of the liver mean dose and of the estimated NTCP were obtained for the healthy part of the liver with the SBRT and IMPT plans.

Studies of partial liver irradiation [4], have shown that the tolerance of the healthy part of the liver is correlated with the liver mean dose. The risk of RILD has been shown to increase rapidly with increasing mean liver dose above the threshold value of 32 Gy. In this study, for all patient cases whose liver mean dose was above this threshold value (7 patients for SBRT and 3 patients for IMPT) the estimated risk for RILD was higher than 5% (Table 3). Because the liver is an organ with parallel organization, high doses given to a limited volume can be tolerated and the risk of toxicity is dominated by the average dose given to the whole organ. Therefore, the liver could benefit from the reduction of the integral dose, which is possible with IMPT.

The sparing of the healthy part of the liver is of utmost importance for patients with primary liver cancer, which in most of the cases have liver cirrhosis of different grades. For these patients, radiotherapy often leads to migration to higher grades of cirrhosis [15], which ultimately can lead to liver failure and death [2]. Proton therapy could appear to be the treatment of choice for patients with a reduced liver function and for postoperative treatment of patients with a reduced total liver volume. In order to fully exploit the potential of PBT for this patient group, functional imaging techniques, e.g., magnetic resonance imaging, can be used to evaluate the liver function prior to the treatment planning. Based on the information obtained in the functional imaging, IMPT plans can be designed in which the beams pass through the parts of the liver which were found to have a reduced liver function.

Clinical follow-up studies have shown that the frequency of RILD occurring after photon beam therapy of liver tumours is low, but that the consequences can be severe. In a follow-up study (median follow-up time of 12.9 months) by Romero et al. [16], it was reported that none of the 34 liver metastases patients developed RILD after being treated with SBRT. In another study conducted by Liu et al. [17], which involved 62 patients with primary and metastatic liver tumours (median follow-up time of 18 months) treated with SBRT, none of the patients with metastatic disease developed RILD, but RILD was diagnosed in one patient with primary liver cancer (Child-Pugh B). One case of death due to liver failure was registered in a follow-up study of colorectal liver-metastases patients (median follow-up time of 4.3 years) who received SBRT [18].

The use of IMPT in the management of tumour sites influenced by organ motion, e.g., the liver, is currently challenging. In IMPT, the individual fields are optimised separately resulting in inhomogeneous dose distributions, which are combined among all the fields included to reach a certain overall dose distribution across the target volume. For SBRT treatments, this overall dose distribution presents large dose gradients. The delivery of high target doses with IMPT, while sparing the OARs, demands that planning and treatment-delivery strategies are adopted to account for the proton-specific and patient-related uncertainties. If not accounted for, these uncertainties may cause changes in the dose distributions produced by the proton beams which may result in a loss of the potential advantages of IMPT. In photon-beam based SBRT of liver tumours, the impact of organ motion are generally minimized by using adequate CTV to PTV margins combined with adequate means of motion mitigation, such as abdominal compression, which was used if required for the patient group included in this study. For IMPT, however, the conventional PTV-based plan optimisation has been shown inappropriate. The use of CTV-based robust optimization allows for a safer delivery of PBT in the presence of proton-specific uncertainties [19]. However, for IMPT-based radiosurgery, there is still no generally accepted standard method for performing a robust optimisation. To create the inhomogeneous dose distribution used in extra-cranial radiosurgery treatments, a robust optimisation which includes nominal objectives for the PTV should be performed. This strategy augmented the CI for the PTV in the present study, as discussed in the study on selective robust optimisation conducted by Li et al. [9]. Despite this fact, the potential of IMPT to reduce the mean doses given to the healthy part of the liver and the estimated NTCP for RILD was still observed.

Another issue of concern in IMPT of moving targets is the so-called interplay effect. In the present study, the planning for IMPT was performed on static CT sets, in which organ motion amplitude was reduced by means of abdominal compression. Even though the impact of organ motion on the dose distributions produced in IMPT are partly reduced with the use of robust optimisation [20, 21], a more thorough analysis of its importance would be valuable. A review of the effects of intra-fraction organ motion and their mitigation strategies have been published by Bert and Durante [22]. The uncertainties introduced by organ motion can be reduced in the clinic by using different motion-mitigation strategies, which include among others, the use of abdominal compression, tumour tracking, gated delivery, re-scanning and the use of larger spot sizes.

In most of the reports presenting comparative studies of photon- and proton-beam therapy, which have been based on the use of radiobiological models [23–25], it has been assumed that the biological-model parameters, obtained in follow-up studies of photon-beam treatments, are also valid for the proton-beam treatments. The same assumption was made in the present work. The NTCP-model parameters used will be updated at a later stage with the clinically observed outcomes following proton therapy. Other sources of uncertainties should also be considered prior to the clinical implementation of the NTCP model-based approach for patient selection. These include the uncertainties in the radiobiological model parameters [26], the inter-patient variation in radiosensitivity [26, 27] and the reduction of the actual three-dimensional dose distributions to DVHs [26]. Furthermore, the DVHs are calculated without taking into account the daily spatial variation in the dose distribution delivered to the patient [28]. A dependence of the estimated NTCP values on the algorithms used for the dose calculations has also been reported [28]. Therefore, the calculated NTCP values must at this point be used with caution for predicting the outcome of radiation treatments.

The patients included in this study have been indicated for palliative treatment with photon-beam based SBRT. The advances in the search of predictive markers for liver metastases [29] could create the possibility to treat curatively patients that were previously considered only for palliative treatment. Moreover, the underlying biological processes at the cellular level, such as enhanced anti-tumour immune response, triggered by the high-dose of SBRT treatments have recently attracted interest [30].

## Conclusions

In this work we found that IMPT could provide a significant reduction of the risk for RILD for most of the patients studied. The successful clinical implementation of an NTCP-model based approach for selection of liver metastases patients requires that a patient data base with follow-up information on the occurrence of side effects, following photon- and proton-beam radiosurgery, is gathered and continuously updated.

## Abbreviations

3D-CRT: Three-dimensional conformal radiation therapy
4D-CT: Four-dimensional computed tomography
CI: Conformity index
CT: Computed tomography
CTV: Clinical target volume
DVH: Dose volume histogram
IBA: Ion Beam Applications
IMPT: Intensity-modulated proton therapy
LKB: Lyman-Kutcher-Burman
LQ: Linear-quadratic
NTCP: Normal tissue complication probability
OAR: Organ at risk
PBT: Proton beam therapy
PTV: Planning target volume
RBE: Relative biological effectiveness
RILD: Radiation-induced liver disease
SBRT: Stereotactic body radiation therapy
TPS: Treatment planning system
VMAT: Volumetric-modulated arc therapy
